# *In Vivo*, Multimodal Imaging of B Cell Distribution and Response to Antibody Immunotherapy in Mice

**DOI:** 10.1371/journal.pone.0010655

**Published:** 2010-05-17

**Authors:** Daniel L. J. Thorek, Patricia Y. Tsao, Vaishali Arora, Lanlan Zhou, Robert A. Eisenberg, Andrew Tsourkas

**Affiliations:** 1 Department of Bioengineering, School of Engineering and Applied Science, University of Pennsylvania, Philadelphia, Pennsylvania, United States of America; 2 Department of Medicine, School of Medicine, University of Pennsylvania, Philadelphia, Pennsylvania, United States of America; New York University, United States of America

## Abstract

**Background:**

B cell depletion immunotherapy has been successfully employed to treat non-Hodgkin's lymphoma. In recent years, increasing attention has been directed towards also using B-cell depletion therapy as a treatment option in autoimmune disorders. However, it appears that the further development of these approaches will depend on a methodology to determine the relation of B-cell depletion to clinical response and how individual patients should be dosed. Thus far, patients have generally been followed by quantification of peripheral blood B cells, but it is not apparent that this measurement accurately reflects systemic B cell dynamics.

**Methodology/Principal Findings:**

Cellular imaging of the targeted population *in vivo* may provide significant insight towards effective therapy and a greater understanding of underlying disease mechanics. Superparamagnetic iron oxide (SPIO) nanoparticles in concert with near infrared (NIR) fluorescent dyes were used to label and track primary C57BL/6 B cells. Following antibody mediated B cell depletion (anti-CD79), NIR-only labeled cells were expeditiously cleared from the circulation and spleen. Interestingly, B cells labeled with both SPIO and NIR were not depleted in the spleen.

**Conclusions/Significance:**

Whole body fluorescent tracking of B cells enabled noninvasive, longitudinal imaging of both the distribution and subsequent depletion of B lymphocytes in the spleen. Quantification of depletion revealed a greater than 40% decrease in splenic fluorescent signal-to-background ratio in antibody treated versus control mice. These data suggest that *in vivo* imaging can be used to follow B cell dynamics, but that the labeling method will need to be carefully chosen. SPIO labeling for tracking purposes, generally thought to be benign, appears to interfere with B cell functions and requires further examination.

## Introduction

Immunotherapeutic depletion of B cells is a clinically approved approach for the treatment of non-Hodgkin's lymphoma, a type of cancer derived from lymphocytes [1]. Rituximab, an engineered anti-CD20 monoclonal antibody that targets B cells at most stages of development, functions therapeutically by specifically eradicating CD20-positive lymphocytes from the patient [2]. Its success has led to its application against a range of non-malignant B cell pathogenic diseases. These include IgM-associated polyneuropathy [3], [4], [5], multiple sclerosis [6], dermatomyositis [7], rheumatoid arthritis (RA) [8], [9], relapsing-remitting multiple sclerosis, and systemic lupus erythematosus (SLE) [10], [11], [12]. Controlled studies with rituximab have already demonstrated a reduction of disease activity in RA patients [13], [14], [15], resulting in its clinical approval for treatment of this autoimmune disease. However, rituximab has failed to show clinical efficacy in Phase II and III trials for treatment of primary progressive multiple sclerosis [16] and SLE [17], [18], [19], [20].

In the clinical setting, the effectiveness of depletion is usually followed through quantification of peripheral blood B cells. However, in SLE patients this measure varies widely for a given dose [21], [22], and does not seem to adequately reflect patient response [10], [12]. Appreciation of the biological response to treatment within the lymphoid organs is therefore expected to be beneficial for greater understanding of underlying disease mechanisms and insight towards development of effective therapies [23].

Cellular and molecular imaging techniques can be used non-invasively, quantitatively and repetitively to visualize cell populations in vivo [24]. Previous studies have utilized radioactive [25], fluorescent [26], [27] and bioluminescent imaging (BLI) [28], [29] approaches to investigate lymphocyte distribution. Recently, a BLI transgenic model was used to monitor the effect of rituximab depletion of a transgenic lymphoma model [30]. Cellular imaging may provide a means to assess the biological response to anti-CD20 and other immunotherapeutics, thereby providing insight into the dose-response behavior and efficacy of treatment.

Magnetic resonance (MR) is a powerful diagnostic tool in preclinical and clinical use that provides high resolution and deep tissue anatomical information. Cell tracking via MR imaging has been realized using superparamagnetic iron oxide (SPIO) nanoparticle contrast agents in a variety of cell types and animal disease models [31], [32], [33]. In the present work we have implemented an ex vivo labeling strategy to load B cells with a non-toxic SPIO configuration, previously determined to efficiently label lymphocytes [34], in combination with a non-toxic near infrared (NIR) cell membrane labeling dye [35]. This approach enabled us to utilize, longitudinally, both MR and optical methods to track contrast labeled cells in the spleen, prior to and following administration of B cell depleting antibody.

## Results

### Labeling of primary murine B cells

**Figure 1:**
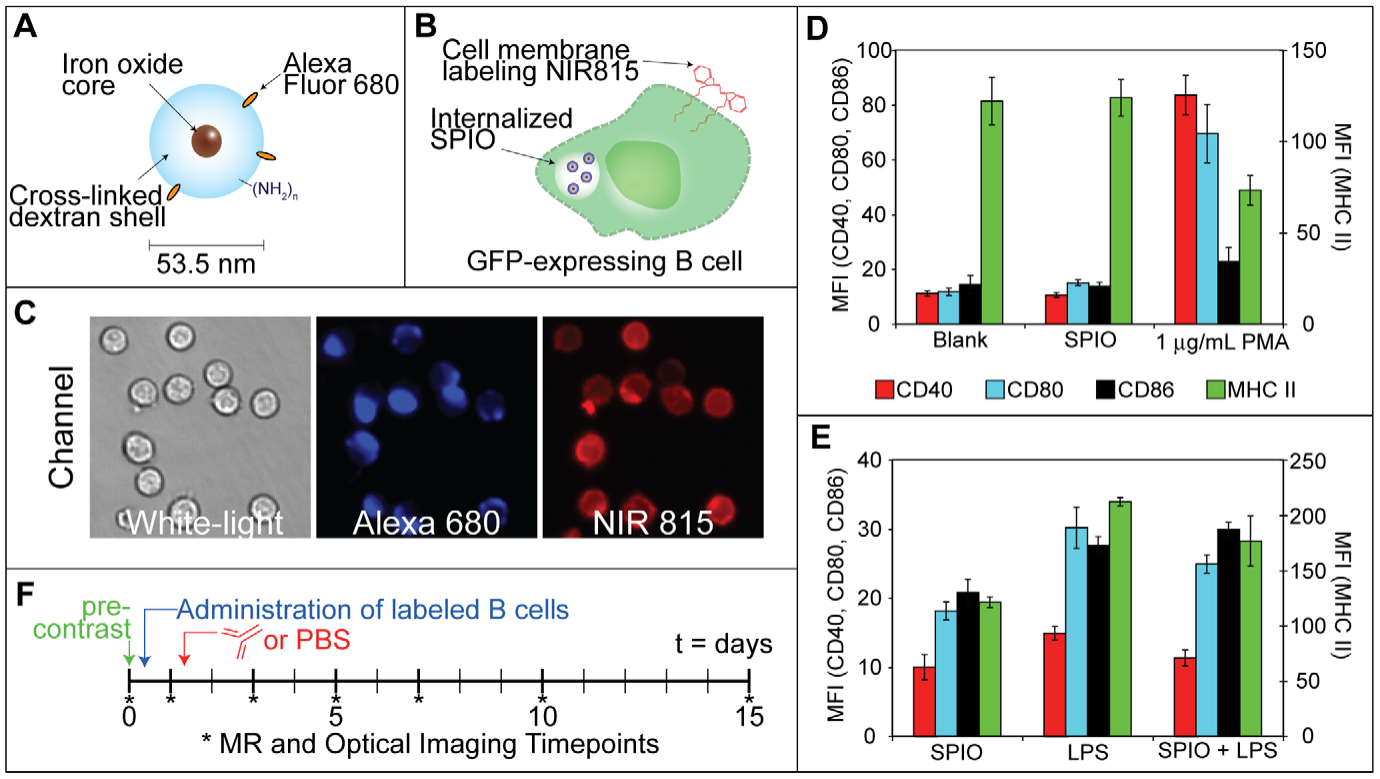
Labeling of B cells with fluorescent and MR tracers and evaluation of cellular response. A, SPIO nanoparticles function as MR contrast agents and consist of dextran-coated iron oxide. The dextran has been aminated and labeled with the dye Alexa 680. B, In addition to SPIO, GFP-expressing B cells are also labeled with the membrane intercalating near infrared dye CellVue NIR815 (NIR815). C, Loading of B cells with SPIO (Alexa 680) and NIR815 was confirmed by fluorescence microscopy (40×). D, SPIO loading did not cause any detectable change in the expression of CD40, CD86, CD80, or MHC II. PMA treated B cells were used as a positive control. E, B cells that were loaded with SPIO could still be activated upon the addition of LPS. F, The contrast labeled cells (20×106) were tail vein injected into C57BL/6 mice on day 0. B cell trafficking and distribution was monitored by MR and optical imaging techniques at the indicated time points. Treatment of either PBS or anti-CD79 was administered following the imaging session on day 1.

The loading of B cells harvested from the spleens of C57BL/6 mice was performed using a cationic 53.5 nm diameter SPIO nanoparticle, schematically illustrated in Figure 1A, through a previously validated procedure [34]. Cells were also labeled with a lipophilic membrane associating dye, CellVue NIR 815 (NIR815), to enable deep tissue fluorescent imaging (Fig. 1B). The proficient loading of the cells was confirmed by fluorescent microscopy, Fig. 1C. B cells from GFP-transgenic mice were employed in order to identify injected cells histologically upon conclusion of in vivo imaging.

Prior to animal experimentation, the expression levels of several cell surface markers were assessed to determine whether SPIO and NIR815-loading led to any change in the cell-activation state. Specifically, the expression levels of CD40, CD80, CD86 and MHC II were evaluated in unlabeled, SPIO-loaded and PMA-activated B cells via flow cytometry, Fig. 1D. No significant change in surface expression was observed between SPIO-labeled and unlabeled cells. PMA, which is known to cause B cell activation [36], resulted in the upregulation of CD40, CD86, and CD80 and the downregulation of MHC II.

The ability of B cells to become activated 24 h after labeling with SPIO was also assessed by monitoring whether treatment with LPS led to cell surface expression patterns that mirrored cells treated with LPS alone. It was found that LPS mediated a similar increase in the expression of CD40, CD80, CD86 and MHC II for both SPIO-labeled and unlabeled B cells (Fig. 1E). These findings suggest that SPIO-loading does not interfere with normal B cell function. Therefore, NIR815-labeled B cells with and without SPIO were administered to C57BL/6 mice and imaged longitudinally to observe the distribution of the B cell population (Table 1).

**Table 1:** Study Groups; Contrast and Treatment.

Group	NIR815	SPIO	Treatment
I	√	√	PBS
Ii	√	√	anti-CD79
Iii	√	Ø	PBS
Iv	√	Ø	anti-CD79

Four groups of animals were imaged. All four groups had B cells administered and were subsequently treated with either anti-CD79 or PBS. The administered B cells were either previously labeled with just a membrane interchelating fluorescent NIR815 dye or co-labeled with both a membrane interchelating dye and SPIO MR contrast, as indicated.

The experimental timeline is illustrated in Fig. 1F. The specific days in which MR and optical imaging were performed are indicated. Animals were treated either with PBS or anti-CD79 antibodies one day following the administration of B cells (immediately after imaging) to determine whether B cell depletion could be directly monitored within the spleen. In addition, one animal from each group (Table 1) was sacrificed and the organs excised for additional analysis.

### Small animal magnetic resonance imaging of cell trafficking

**Figure 2:**
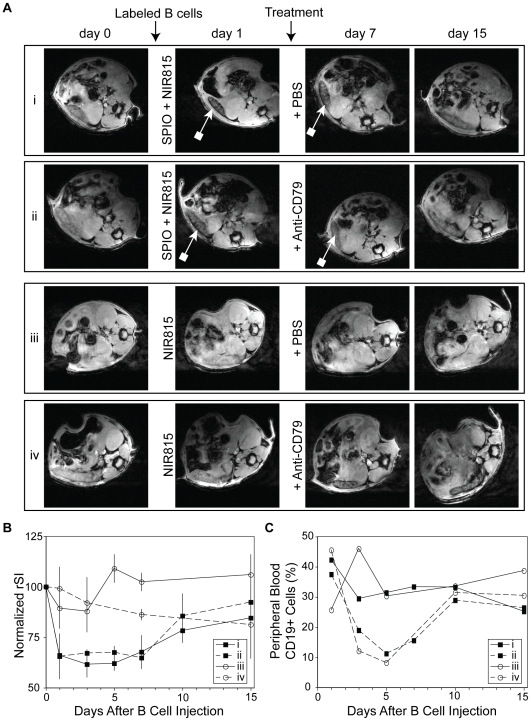
Longitudinal MR imaging and quantification of contrast-labeled B cells prior to and following B cell depletion therapy. A, Representative axial T2*-weighted images of mice either pre-injection (day 0) or on the indicated days following injection of contrast labeled B cells. Hypointensity of the spleen was noted in animals of the groups given SPIO-loaded B cells (block ended arrow: groups i and ii). Isointense signal was observed on all pre-contrast images and in animals injected with NIR815-labeled B cells (groups iii and iv). Either PBS or anti-CD79 was administered to each group after imaging on day 1. B, Relative signal intensity (rSI; calculated as the ratio of signal in the spleen to paraspinal muscle) is plotted, normalized to pre-contrast images. For SPIO-labeled B cell groups (i and ii, ▪) a rapid and pronounced decrease in rSI was observed. There was also only limited difference between the normalized rSI of PBS (i, solid line) and anti-CD79 (ii, dashed line) administered groups following treatment. There was no significant change in spleen signal for groups devoid of SPIO, after B cell injection (iii and iv, ○). A gradual decrease in normalized rSI was seen in the anti-CD79 treated group (iv, dashed line). C, Although SPIO-labeled B cells were not depleted following the administration of anti-CD79 antibodies as detected by MR, the number of peripheral B cells were reduced compared to PBS-treated controls. A similar depletion profile was seen for the NIR-only labeled B cell groups.

MR imaging was used longitudinally to monitor the trafficking and biodistribution of B cells labeled with both SPIO and NIR815 or NIR815 only (i.e. SPIO-free). One day following the administration of SPIO-labeled B cells, the spleen appeared darker on T2*-weighted images (Fig. 2A, row i). This hypointensity is consistent with the accumulation of SPIO. The spleen remained hypointense for several days, but gradually returned to near baseline over the course of two weeks. Animals that were treated with anti-CD79 antibody one day following the administration of SPIO-labeled B cells (Fig. 2A, row ii) exhibited a similar pattern of contrast enhancement as the control (PBS-treated) animals over the course of the study.

There was no apparent signal change in the spleen one day after animals were injected with B cells labeled with just NIR815 dyes (Fig. 2A, row iii). However, animals that were subsequently treated with anti-CD79 antibodies did exhibit a gradual loss in T_2_*-weighted signal within the spleen over the following two weeks (Fig. 2A, row iv). No change in T_2_*-weighted signal was observed in PBS-treated controls over the same time period.

Quantitative measurements of lymphocyte homing to the spleen were determined by measuring the signal intensity within the spleen. All measurements were normalized by the signal intensity of the surrounding muscle to adjust for image variability. The resulting value is referred to as the relative signal intensity (rSI). All rSI values were further normalized by day 0 measurements. The normalized rSI was reduced ∼40% one day following injection of SPIO-loaded B cells, compared with pre-contrast images (Fig. 2B, groups i and ii). Over the following two weeks, the normalized signal ratio gradually increased towards pre-contrast values. There was no statistically significant difference (p<0.05) in normalized rSI between untreated (i) and treated (ii) groups. These findings suggest that SPIO could potentially interfere with the efficacy of B cell depletion therapy. Animals that had SPIO-free B cells (i.e. NIR815 only) administered exhibited a statistically significant difference in the normalized rSI between treated and untreated groups (Fig. 2B, iii and iv) at day 7. There also appeared to be some difference on day 14, but variability of rSI was too great to achieve statistical significance at this time point.

To confirm that B cells were actually being depleted following the administration of anti-CD79 antibody, peripheral B cell counts were acquired from sacrificed animals at each imaging time point. It was found that the B cells were in fact rapidly depleted, reaching a minimum approximately 4 days after the injection of anti-CD79 antibodies (Fig. 2C). Thereafter, the number of peripheral B cells gradually increased, returning to baseline levels approximately 9-14 days after treatment.

### Whole body *in vivo* fluorescent imaging

**Figure 3:**
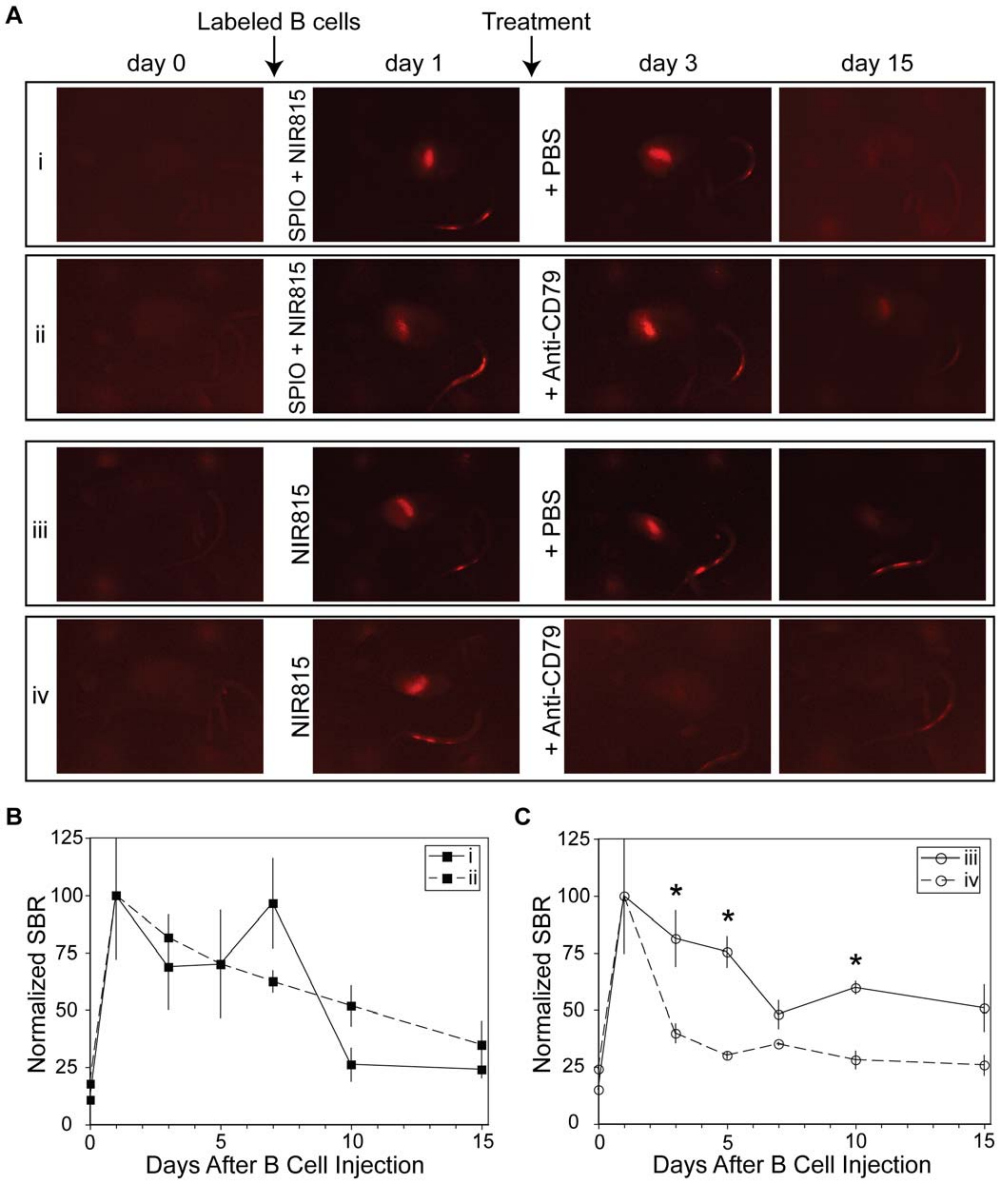
In vivo fluorescent imaging of contrast-labeled B cells prior to and following B cell depletion therapy. A, Representative whole animal fluorescence images of B cells loaded with SPIO and NIR815 (groups i and ii) or just NIR815 (groups iii and iv) following injection into C57BL/6 mice. Prior to injection (day 0), no signal was evident in the NIR channel. Signal accumulated within the spleen by 24 h after the cell injection. Immediately following imaging on day 1, animals were injected with either anti-CD79 antibodies or PBS. Anti-CD79 treatment led to a rapid (by day 3) loss is signal in mice injected with B cells labeled with NIR815 only. B, The abundance of B cells, labeled with SPIO and NIR815, in the spleen was quantified by measuring the spleen-to-muscle signal–to-background ratio (SBR). Quantification of fluorescence revealed only a gradual loss in SBR following treatment with PBS (group I, solid line) and anti-CD79 antibodies (group ii, dashed line). No statistical significance was observed between the two groups (p<0.05). C, NIR815-labeled B cells (i.e. no SPIO) were rapidly depleted following administration of anti-CD79 antibodies (group iv, dashed line) compared with PBS-treated controls (group iii, solid line). Statistical significance for individual time points is indicated with an asterisk (p<0.05).

In addition to MR imaging, it was possible to detect and monitor the trafficking and distribution of the introduced B cell population via NIR fluorescence imaging (Fig. 3A). Acquisitions of both Alexa 680 (i.e. fluorescent label on SPIO) and CellVue NIR815 (membrane dye) were obtained; however, there was insufficient signal from the Alexa 680 dye to be detected above the autofluorescence of living animals (not shown).

Whole body in vivo fluorescent imaging showed that the B cells in all test groups were predominantly localized within the spleen one day after delivery (Fig. 3A). Little fluorescence was seen outside the spleen, except at the site of tail vein injection where presumably a small percentage of cells had leaked subcutaneously. Consistent with MR imaging data, B cells that were labeled with SPIO and NIR815 did not appear depleted following administration of anti-CD79 antibodies (Fig. 3A, row i), compared with PBS treated controls (Fig. 3A, row ii). In both groups, the fluorescent signal within the spleen decreased gradually over the course of the experiment returning to near baseline levels by day 15. In contrast, the rapid depletion of B cells labeled with only NIR815 (i.e. SPIO-free) could be observed via fluorescence imaging (Fig. 3A, rows iii and iv). Within two days of administration of anti-CD79 antibody, the fluorescent signal was reduced to near baseline levels, whereas B cells within PBS-treated control animals exhibited a detectable, although decreasing, fluorescent signal as late as 14 days after the administration of PBS.

The effect of the B cell depleting antibody was quantified through measurement of fluorescent intensity in the spleen relative to the background (SBR), Fig. 3B. These data revealed that when cells were labeled with SPIO and NIR815, there was no statistical difference in the SBR between anti-CD79 antibody- and PBS-treated subjects (Fig. 3B). Conversely, when cells were labeled with NIR815 alone, there was a ∼65% reduction in the SBR two days following the administration of anti-CD79 antibodies compared with only a 20% reduction of SBR in PBS-treated controls (Fig. 3C). The difference in the SBR between treated and untreated groups remained statistically significant for the remainder of the study, 14 days in total.

### *Ex vivo* organ fluorescence

**Figure 4:**
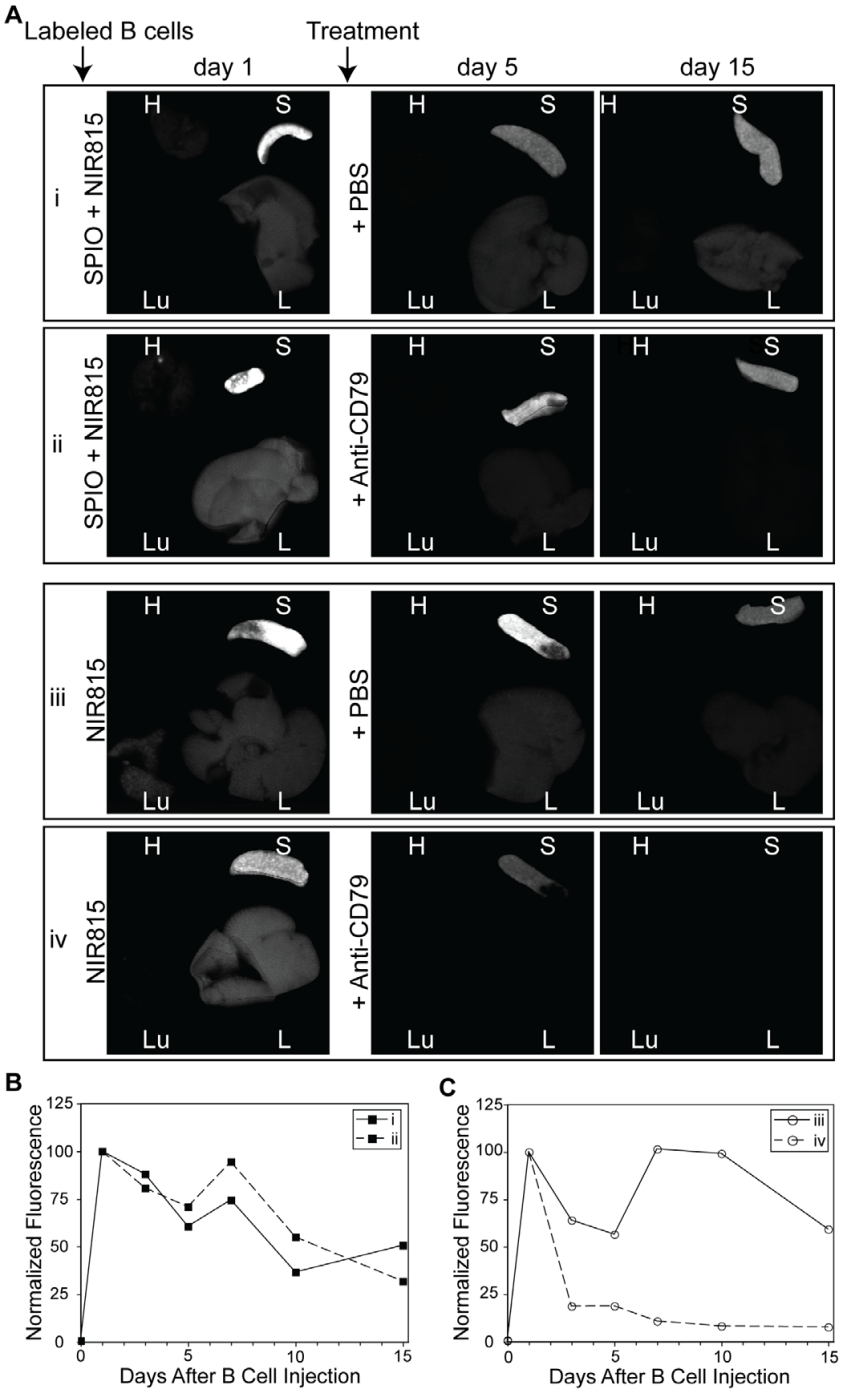
Fluorescent images of contrast-labeled B cells in excised organs. A, Representative fluorescent images of the excised heart (H), lung (L), spleen (S), and liver (L) from animals injected with contrast labeled B cells. The B cells were either labeled with SPIO and NIR815 (groups i and ii) or just NIR815 (groups iii and iv), as indicated. Animals were either treated with PBS (groups i and iii) or anti-CD79 antibodies (ii and iv) following imaging on day 1. One animal per group was sacrificed at each time point, immediately following MR and in vivo fluorescent imaging. B and C, The mean fluorescence intensity (MFI) of the spleen, normalized to day 1 values for each group, is plotted for the length of the experiment. A gradual loss of signal from the spleen is seen in all groups, save the rapid decrease following treatment of NIR815-only labeled cells (iv).

Fluorescent images of the excised liver, lungs, heart and spleen were acquired from a single animal from each group, at each time point (Fig. 4A). The distribution of B cells as determined by ex vivo fluorescence imaging confirmed that homing to the spleen was rapid. One day after B cell injection a strong signal was observed in the spleen. Significantly less fluorescence was observed in the liver and little to no signal was detectable in the heart or lungs. Consistent with live animals imaging studies, SPIO seemed to interfere with the ability to deplete the transferred cells as there was no significant difference between control and treated mice (Fig. 4B). However, in the absence of SPIO, B cell depletion could be readily observed through the loss of NIR815 fluorescence in the spleen. Quantitative analysis revealed a ∼80% reduction in the mean fluorescence intensity (MFI) of NIR815 within the spleen only two days after the administration of anti-CD79 antibodies (Fig. 4C). Mice treated with PBS only exhibited a ∼35% reduction in MFI at the same time point.

### Histology

**Figure 5:**
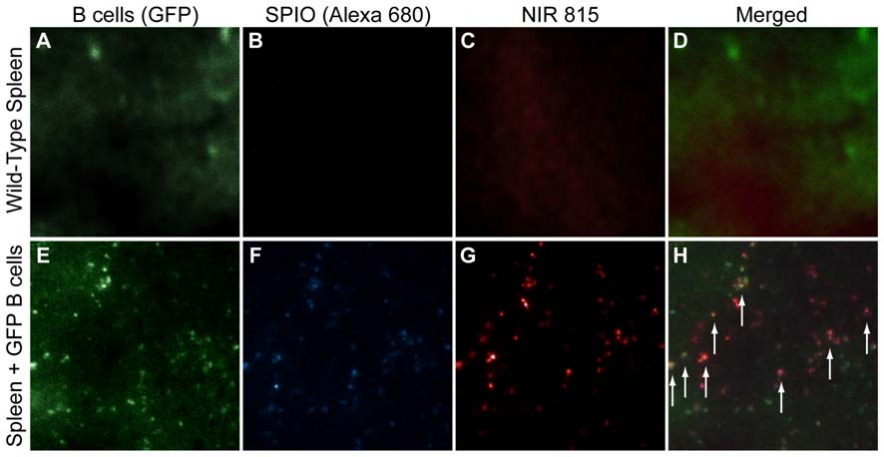
Fluorescent images of spleens following histological sectioning. (A–D) Representative fluorescent images of an excised and sectioned spleen, obtained from a mouse that was not subject to B cell transfer. (A) Aside from autofluorescence, there is little detectable fluorescent signal in the (A) GFP, (B) Alexa-680 (i.e. SPIO), and (C) NIR815 channels. (D) A composite image shows no significant co-localization between the fluo rescent images. (E–F) Representative images of a spleen that was excised and sectioned 7 days following the transfer of SPIO- and NIR815-labeled B cells into C57BL/6 mice. (E) Despite the high level of autofluorescence, distinct punctate areas of fluorescence could still be discerned. Presumably, these fluorescent signals signify the presence of GFP-positive B cells. Similar patterns of punctate fluorescent signals were also observed in the (F) SPIO and (G) NIR815 channels. (H) The composite image shows that there is considerable overlap between the fluorescent signals in all three channels (arrows). All images were acquired using a LUC PLAN FLN 40× objective (NA 0.6).

Immediately following ex vivo fluorescent imaging of the excised organs, the spleens were sectioned and imaged by fluorescence microscopy (Fig. 5). In animals that had been injected with SPIO-labeled B cells, there was a clear co-localization between the GFP-positive B cells, Alexa-680 (i.e. fluorescent label on SPIO), and NIR815. In contrast, in animals where no B cells were administered, only low levels of autofluorescence were apparent. These results provide strong evidence that the SPIO and NIR815 signals reflect the actual B cell biodistribution, i.e. the contrast agents are not transferred to other cells over the course of the experiment.

## Discussion

There currently exists both an academic and clinical deficit in the techniques available to monitor B cell depletion quantitatively within organs. This manifests as a significant problem considering the high degree of variability in the response of patients to rituximab treatment [37], [38], [39], [40]. Critically, this also hinders development of novel therapeutics for B cell dysfunction-dependent diseases resistant to anti-CD20 treatment, such as SLE [23]. The evaluation of treatment efficacy and correlation to clinical outcome can only be determined under fixed time-point, ex vivo methodologies or through peripheral blood B cell measurements of limited value. Several bioluminescent imaging approaches to visualize treatment of B cell lymphoma have been described; however these non-translatable strategies have utilized chimeric mouse models to enable the evaluation of human antibody targeting [30]. In the present study we have evaluated the ability of SPIO and NIR815 membrane dyes to enable in vivo observation of B cell distribution and response to antibody mediated depletion using MR and fluorescent imaging.

Cationic SPIO were efficiently internalized by isolated primary B cells during a simple 2 h incubation period. Internalization did not significantly alter morphology, lead to clumping, or affect the surface expression patterns of isolated B cells as assessed by the measurement of CD40, CD80, CD86 and MHC II expression by immunostaining and flow cytometry. Further, SPIO did not appear to interfere with the ability of isolated B cells to become activated upon treatment with LPS. These findings led us to initially believe that SPIO were essentially intracellularly inert after loading, with no detectable impact on the functionality of B cells.

Following the transfer of SPIO-labeled B cells into C57BL/6 mice, a dramatic reduction in MR signal intensity was observed within the spleen on T_2_*-weighted images, consistent with the accumulation of iron oxide nanoparticles. Homing to the spleen was also observed through fluorescence imaging of NIR815 in both live animals and excised organs. However, administration of anti-CD79 antibodies did not result in any significant splenic depletion of the SPIO-labeled B cells in recipient C57BL/6 mice compared with PBS-treated control animals. This was verified through MR imaging as well as optical imaging of live animals and excised organs.

To ensure that the administered anti-CD79 antibodies were capable of effectively depleting B cells, peripheral B cell counts were acquired over the course of the study. As expected, the endogenous B cells were rapidly depleted from circulation. It should be noted that the depletion of peripheral B cells has previously been shown to correlate with the depletion of B cells within the spleen of healthy animals [41], [42]. These findings suggest, in contradistinction to the in vitro B cell functionality and expression assays and the apparently normal B cell trafficking to the spleen, that SPIO-labeling does interfere with B cell regulation in vivo. The interference was SPIO-specific as B cells that were subject to isolation and labeling with just the membrane dye could be depleted with anti-CD79 antibodies following their transfer into C57BL/6 mice. The same protocol was followed in both studies, outside of the additional 2 h incubation with SPIO. Therefore, SPIO seem to have subtle effects on B cell function that could easily be overlooked. This is a particularly important finding when considering the use of SPIO for cell tracking applications. Numerous, pre-clinical studies have already been conducted illustrating the ability to monitor cell distribution via MR, including applications involving dendritic cells, T-lymphocytes, natural killer (NK) cells, macrophages and stem cells [43], [44], [45], [46], [47]. However, very few of these studies look at downstream functionality beyond cell homing. Our findings suggest that cell trafficking alone may not be a sufficient marker for normal cell behavior and that caution must be taken when applying these methods.

Recently, several studies have also found that adverse cellular effects may result from iron oxide particle labeling [48], or that SPIO may not be suitable for long term in vivo visualization [49]. The current system in which both a fluorescent cell associated dye and SPIO have been used has the potential to provide insight into the interfering effects of nanoparticulate contrast media.

Despite the shortcomings of SPIO-labeling in this study, B cells that were subject to labeling with just the NIR815 membrane dye could be effectively depleted with anti-CD79 antibody following transfer into C57BL/6 mice. The whole body fluorescent tracking of this population of B cells enabled direct, longitudinal and quantitative examination of both the initial accumulation and subsequent depletion of the B lymphocytes in the spleen. We believe that this imaging approach can be extended to animal models of autoimmune disease and will allow greater understand of the efficacy and physiological impact of various depletion therapies.

## Materials and Methods

All chemicals and equipment were purchased from Fisher Scientific (Pittsburgh, PA), unless otherwise noted. Mice (Jackson Laboratories; Bar Harbor, ME) were housed under USDA- and AAALAC-approved conditions with free access to food and water. The University of Pennsylvania Institutional Animal Care and Use Committee and Small Animal Imaging Facility (SAIF) Animal Oversight Committee approved all experimental procedures. All in vivo imaging was conducted at the SAIF in the Department of Radiology at the University of Pennsylvania.

### Nanoparticle Synthesis and Modification

SPIO, 53.5 nm hydrodynamic diameter, R_2_ of 82 mM^−1^sec^−1^, were produced as previously described [34]. Briefly, iron chloride salts (4 g of FeCl_3_ and 1.5 g of FeCl_2_) were chemically co-precipitated through base addition in inert atmosphere at 4°C in the presence of dextran-T10 (25 g, GE Healthcare; Waukesha, WI). The solution was heated to 90°C for 1 h and then ultracentrifuged at 20 k RCF. SPIO were purified from reactants via diafiltration (100 kDa MWCO; GE Healthcare) against 0.02 M citrate buffer.

The dextran coating was cross-linked in base (25% v/v 10 M NaOH) with epichlorohydrin (33%) for 24 h. Subsequently, the dextran was aminated by adding NH_4_OH to a volume fraction of 25%. The reaction was allowed to proceed for another 24 h. Again, particles were diafiltrated from excess reactants. The resulting particles were reacted at a 1∶10 molar ratio of SPIO:Alexa Fluor 680 (Invitrogen; Carlsbad, CA) in pH 9 sodium bicarbonate buffer. The labeled particles were purified on a PD10 gel filtration column in PBS to yield approximately 3 dye molecules per particle.

### Cell Labeling

Splenocytes were obtained from C57BL/6 or C57BL/6-Tg(UBC-GFP)30Scha/J mice, as required. B cells were purified using red blood cell lysing buffer and anti-CD43 MACS beads (Miltenyi Biotec; Hamburg, Germany) for negative selection [50]. B cells were incubated with nanoparticles (50 µg/mL) in fully supplemented RPMI 1640 (ATCC; Manassas, VA) for 2 h. The cells were washed of non-internalized SPIO and labeled with a 1/50 dilution of CellVue® NIR815 dye (NIR815, Molecular Targeting Technologies; Malvern, PA) following manufacturer instructions. For NIR815-only labeled cells, the SPIO labeling step was omitted.

### Animal Procedures

Four groups of 8 adult (8 week old) male C57BL/6 mice were placed on AIN-76A low-autofluorescence rodent diet (Research Diets, Inc.; New Brunswick, NJ). Six days after food change, animals were tail vein injected with 20 M primary B cells derived from GFP-transgenic mice. Two groups were injected with SPIO and NIR815-labeled cells, and two with cells that were labeled with the NIR815 dye alone; Table 1. Three subjects per group were imaged using MR and whole-body fluorescent imaging at each of the following time points; prior to administration, 1, 3, 5, 7, 10 and 15 days post-injection.

Immediately following the day 1 imaging time point, an intraperitoneal injection (IP) of either 200 µg of anti-CD79β (MH79-16, Armenian hamster IgG [42], [51]) or PBS was given. Peripheral blood lymphocytes (PBL) were measured, and the heart, liver, lungs and spleen were removed for ex vivo fluorescent organ imaging (Odyssey, LiCOR; Lincoln, NE) from one mouse per group at each time point. PBL were purified of red blood cells with ACK and stained with anti-CD19-FITC (1d3, rIgG). The cells were subsequently fixed with 4% formaldehyde and PBL counts were measured by flow cytometry (Easycyte, Guava Technologies, Inc; Hayward, CA).

### Cell Activation

Activation of primary C57BL/6 B cells or splenocytes by incubation with SPIO, phorbol 12-myristate 13-acetate (PMA, Sigma Aldrich; St. Louis, MO) and lipopolysaccharide (LPS - E. Coli 055:B5, Sigma) was assessed by flow cytometry. B cells were incubated with SPIO (as above), washed and maintained in culture media for 24 h. As a positive control for activation, unlabeled B cells were incubated with LPS (0.01 µg/mL) for 24 h. B cells were also assessed for their ability to activate after loading with SPIO by adding LPS to the culturing medium of SPIO-labeled cells and incubating for 24 h. Activation was measured by changes in surface marker expression: antibodies (1/200) against CD40 (PE), CD86 (PE), CD80 (FITC) and MHCII (FITC) were used to label cells after triplicate washing from culture media into 0.1% BSA in PBS. To prevent internalization of antibody, labeling was conducted on ice.

### *In Vivo* Fluorescence Imaging

Mice were induced with inhalation anesthesia, using in a 4% mixture of Isoflurane in oxygen. Mice were maintained at a 2% mixture of the gas and shaved on their left side. 2-2-2 Tribromoethanol (Avertin; 500 mg/kg dose; approximately 200 µL) was administered IP 10 minutes before imaging to keep animals motionless during acquisition. Spectral fluorescence images of recumbent mice were acquired using a Maestro fluorescence imaging system (CRi; Woburn, MA). The red filter set (excitation range 615 to 665 nm; emission, 700 nm longpass) was used to detect Alexa680-SPIO and the near infrared filter set (excitation range 710 to 760 nm; emission, 800 nm longpass) enabled detection of the cell membrane bound NIR815. Each spectral image set was acquired using a 5 sec exposure with acquisition at 10 nm steps through the emission range. The spectral fluorescence images consisted of data from the two dyes. The autofluorescence spectra were then unmixed based on their spectral patterns using commercial software (Maestro software, CRi). Line intensities were generated through the long axis of the spleen. Mean intensities of the signal at this organ and background were computed and averaged for each image.

### *In Vivo* MR Imaging and Analysis

Magnetic resonance imaging was conducted using a horizontal Varian 9.4 T small animal imaging system (Varian; Palo Alto, CA). Gradient coils were upgraded during the course of the study, going from a diameter of 14 to 12 cm and a gradient strength of 10 G/cm to 25 G/cm (both Varian). A dual coil, actively detuned system was employed; a 70 mm receiver volume coil was paired with a 2.5 cm surface coil (InsightMRI, LLC; Worcester, MA). Mice were induced using inhalation anesthesia, 4% isoflurane, and maintained for the duration of image acquisition with 2% isoflurane. The animals were placed on their right side in a split top mouse chamber sled (m2m Imaging; Cleveland, OH) affixed to a custom built poly-(methyl methacrylate) patient bed. The surface coil was applied to the left side of the animal (proximal to the spleen) and fixed in position using surgical tape to both the mouse and bed. Temperature and electrocardiography probes were used to monitor the mice in the 37°C environment supplied by an air heating system (SA Instruments, Inc.; Stony Brook, NY).

Scout scans were used to identify the anatomy of interest. Following recognition of the spleen, axial sections (1 mm thick) were acquired in a 36×36 mm field of view. Acquisition sets consisted of np = 256, nv = 256, under T_2_*-weighted parameters. A gradient echo multislice sequence was used with 2 averages, and TR/TE 200/6 msec. Images were analyzed by defining a ROI for the spleen and background (paraspinal) muscle. The relative intensity of the spleen was then calculated, and statistical significance for the image analysis was assessed using a two-tailed parametric analysis. A p-value<0.05 was used to represent statistical significance.

### Fluorescence Microscopy

Fluorescence microscopy was performed using an Olympus IX 81 motorized inverted fluorescence microscope equipped with an Ixon (Andor Technologies; Belfast, N. Ireland) monochrome digital camera, an X-Cite 120 excitation source (EXFO; Quebec, PQ) and Sutter excitation and emission filter wheels (Novato, CA). Images were acquired using a LUC PLAN FLN 40× objective (NA 0.6) with Alexa 680 and NIR815 filter sets, (HQ665/45, Q695LP, HQ725/50) and (HQ710/75, Q750LP, HQ810/90), respectively (Chroma Technology Corp.; Burlington, VT).

### Histology

Immediately following ex vivo fluorescent imaging of the excised organs, spleens were embedded in Optimal Cutting Temperature compound (Tissue-Tek, Sakura Finetek Americas, Inc.; Torrance, CA). Fresh cut sections (8 µm thickness) were air dried for 1 hr and then sealed under coverslips with cyanoacrylate ester glue. Tissue was imaged for GFP (HQ480/40×, Q505LP, HQ535/50 m), Alexa 680 and NIR815 by fluorescence microscopy, as above.

### Statistics

All data are presented as mean ± standard error. For statistical evaluation, a two-tailed Student's t-test was used, and *p*<0.05 was considered significant. Calculations were performed using a commercially available plotting and statistics software package (GraphPad Prism; GraphPad Software, San Diego, CA).
